# What is the Impact of Negative Pressure Wound Therapy on Healing in Patients Post Excision of Pilonidal Sinus? A Systematic Review and Meta‐Analysis

**DOI:** 10.1111/iwj.70194

**Published:** 2025-04-27

**Authors:** Liliana Patricia Ferreira Morais, Zena Elizabeth Helen Moore, Declan Patton, Tom O' Connor, Pinar Avsar, Hannah Jane Elizabeth Wilson

**Affiliations:** ^1^ Royal College of Surgeons in Ireland (RCSI) University of Medicine and Health Sciences Dublin Ireland; ^2^ School of Nursing and Midwifery RCSI University of Medicine and Health Sciences Dublin Ireland; ^3^ Skin Wounds and Trauma Research Centre RCSI University of Medicine and Health Sciences Dublin Ireland; ^4^ Fakeeh College of Health Sciences Jeddah Saudi Arabia; ^5^ School of Nursing and Midwifery Griffith University Brisbane Queensland Australia; ^6^ Department of Public Health Faculty of Medicine and Health Science Ghent University Shanghai China; ^7^ National Health and Medical Research Council Centre of Research Excellence in Wiser Wound Care Menzies Health Institute Queensland Brisbane Queensland Australia; ^8^ Honorary Visiting Professor Cardiff University Cardiff, Wales UK; ^9^ Honorary Senior Fellow, Faculty of Science, Medicine and Health University of Wollongong Wollongong New South Wales Australia; ^10^ Lida Institute Shanghai China

**Keywords:** negative pressure wound therapy, NPWT, pilonidal cyst, pilonidal sinus, systematic review

## Abstract

This systematic review aimed to determine the impact of negative pressure wound therapy on healing in patients with pilonidal sinus following surgical excision. Using systematic review methodology, we included original research studies written in English. The search was conducted using CINAHL Plus, Ovid, PubMed, EBSCO Host and Cochrane databases. Quality appraisal was undertaken using the Cochrane Collaboration tool for assessing risk of bias and the Grading of Recommendations Assessment, Development and Evaluation (GRADE). Data were analysed using meta‐analysis where appropriate; otherwise, the data are presented narratively. Ten studies were included with a mean sample size of 61 (SD: 33). Three studies were randomised control trials, four were retrospective studies, two were prospective cohort studies and one was pre‐post study. Studies showed reduction in healing time, recurrence rates and postoperative pain, as well as higher patients' satisfaction with the use negative pressure wound therapy, however, the certainty of the evidence was very low. The evidence for the intervention is uncertain thus, it is unclear whether NPWT makes any difference when compared to any other dressings, on time to healing, recurrence, pain or patient's experience. Further high‐quality research with larger sample sizes is recommended to clearly explore the impact of NPWT on PS healing, recurrence, pain and patient's satisfaction.

**Summary**

- Pilonidal Sinus Disease is a debilitating chronic inflammatory disease, and the management of pilonidal sinus wounds post‐surgical intervention can be very challenging.
- Negative pressure wound therapy has demonstrated its effectiveness with a wide range of complex surgical wounds by promoting healing, decreasing bacterial contamination, managing exudate and oedema.
- Data showed very low certainty of evidence, demanding more high‐quality research with larger sample sizes to explore the impact of NPWT on PS healing, recurrence, pain and patient's satisfaction.

## 1 Introduction

Pilonidal sinus disease (PSD) is a common and debilitating chronic inflammatory disease affecting 0.7% of the population [1]. This condition refers to inflammation of the skin and subcutaneous tissue, which can occur in any part of the body but, most commonly in the sacrococcygeal area [2]. PSD may have a variety of presentations, ranging from a simple cyst to multiple sinuses and may include asymptomatic to painful and swollen draining lesions [3].

There are several surgical techniques used as primary interventions for the treatment of acute abscesses, yet consensus on the optimal approach remains elusive [4]. Despite the chosen surgical approach, the aim should be the complete excision of the affected tissue, followed by the most appropriate wound healing approach [5]. The impact of the disease extends beyond quality of life to financial considerations, as recurrence rates are high and patients often require multiple treatment interventions, leading to increased healthcare cost [6].

The management of pilonidal sinus (PS) wounds post‐surgical intervention can be very challenging, due to their depth, size, susceptibility to infection and large amounts of exudate [1]. An ideal dressing should conform to the wound's shape and size, absorb excess discharge, regulate temperature and moisture, prevent bacterial infiltration, apply suitable pressure, minimise pain, aid in debridement and epithelialization and be cost‐effective by reducing the need for frequent replacement [7].

With regards to wound management options, negative pressure wound therapy (NPWT) has demonstrated its effectiveness with a wide range of complex surgical wounds [1]. NPWT is a closed and sealed dressing system connected to a vacuum pump by a suction tube, which creates negative pressure to the wound, collecting the wound exudate into a waste collector [8]. Evidence suggests that NPWT promotes wound healing by enhancing local blood flow, stimulating granulating tissue, decreasing bacterial contamination, exudate and oedema [8]. NPWT is being used frequently on closed incisional wounds prophylactically, as well as on wounds healing by secondary intention [9].

### 1.1 Aim

NPWT has been used as a treatment regimen for PS wounds for many years [10]. However, the authors found many studies but no systematic review on the topic. Thus, the aim of this systematic review was to determine the impact of negative pressure wound therapy on healing in patients with pilonidal sinus following surgical excision.

## 2 Materials and Methods

### 2.1 Design

A systematic review of the published literature was conducted, following the recommendations and guidelines outlined in the Cochrane Handbook for Systematic Reviews of Interventions [11]. Additionally, the principles of the Preferred Reporting Items for Systematic Reviews and Meta‐Analyses (PRISMA) were adhered to [12]. The study question was developed using a structured approach referred to as PICO (population, intervention, comparator and outcomes). The specific elements of PICO were as follows:

- Population: Patients post excision of PS;
- Intervention: NPWT;
- Comparison: Any other dressings;
- Outcome: Primary: wound healing; Secondary: recurrence; pain and patient’ experience.

### 2.2 Inclusion and Exclusion Criteria

The inclusion criteria were as follows:

- Patients undergoing PS (pilonidal sinus) surgical excision;
- Patients receiving NPWT (negative pressure wound therapy) for the treatment of PS surgical wounds healing by primary or secondary intention;
- Studies published in English;
- Studies with a comparative design (e.g., randomised controlled trials, cohort studies and case–control studies) and other relevant study designs providing data on outcomes of interest.

The exclusion criteria were as follows:

- Patients with PS surgical wounds not receiving NPWT;
- Patients with non‐PS surgical wounds receiving NPWT;
- Studies published in languages other than English;
- Case reports, case series, editorials or commentaries.

### 2.3 Electronic Searches

The following databases were searched from December 2023 to January 2024: PubMed, CINAHL Plus, Ovid, Cochrane and EBSCO Host. The PubMed search strategy was as follows: (Negative pressure wound therapy) OR (NPWT) OR (V.A.C. Therapy) OR (Vacuum‐Assisted Closure) OR (PICO) OR (Prevena) AND (Pilonidal sinus disease) OR (Pilonidal Sinus) OR (Pilonidal Wound) OR (Pilonidal Cyst) OR (Sacrococcygeal Cyst) OR (Sacrococcygeal Sinus). This search was adapted to other online databases according to the syntax in each database. To identify further published, unpublished and ongoing studies, this systematic review:

- Scanned reference lists of all identified studies and reviews to assess for further relevant citations.
- Performed a manual search other relevant literature, to enhance the capture of relevant and unique literature (ClinicalTrials.gov, Google scholar, ResearchGate).
- Searched conference proceedings, research reports and dissertations.

### 2.4 Study Selection

Article titles were assessed by two authors independently, and the abstracts of the studies (when available) identified by the search strategy were screened for their eligibility, according to the inclusion and exclusion criteria. The full‐text versions of potentially relevant studies were obtained, and the same two authors independently screened these against the inclusion criteria. Consensus between the two authors in relation to the studies and the data to be included was obtained through a discussion when discrepancies were identified.

### 2.5 Data Extraction

Data from the included articles were extracted and entered into a pre‐designed table using the following headings: study name, author and date of the study, setting, sample size, design, outcomes (wound healing, recurrence, pain and patients' experience) as defined by the study's authors and limitations. Two authors undertook this independently.

### 2.6 Data Analysis

Following data extraction both a narrative analysis and meta‐analysis statistical synthesis was considered appropriate. First, the data were narratively summarised, giving an overview of the study setting, geographical location, study settings, sample sizes and primary and secondary outcomes. Data are presented using means and standard deviations to depict the data obtained. Meta‐analysis statistical synthesis was undertaken using RevMan [13]. Relative risks (RRs) and 95% confidence intervals (CI) were calculated for dichotomous outcomes.

#### 2.6.1 Assessment of Heterogeneity

Results of comparable trials were pooled using either a fixed‐effect model or random effects model, depending on heterogeneity which was investigated using the I^2^ [statistic. Heterogeneity was assessed using the I^2^ [ test [14] Where there was evidence of substantial heterogeneity (*I* ^2^ > 50%), we used a random effects model in the meta‐analysis. In the absence of significant heterogeneity, a fixed effects model was employed in the meta‐analysis [14].

#### 2.6.2 Dealing With Missing Data

If data had been missing, we would have reached out to the study's authors. However, if we deemed the data to be missing at random, we proceeded with the analysis using the available information. For missing outcome data, we conducted an available‐case analysis, relying on the number of participants with known outcome data [11].

### 2.7 Quality Appraisal

**FIGURE 1:**
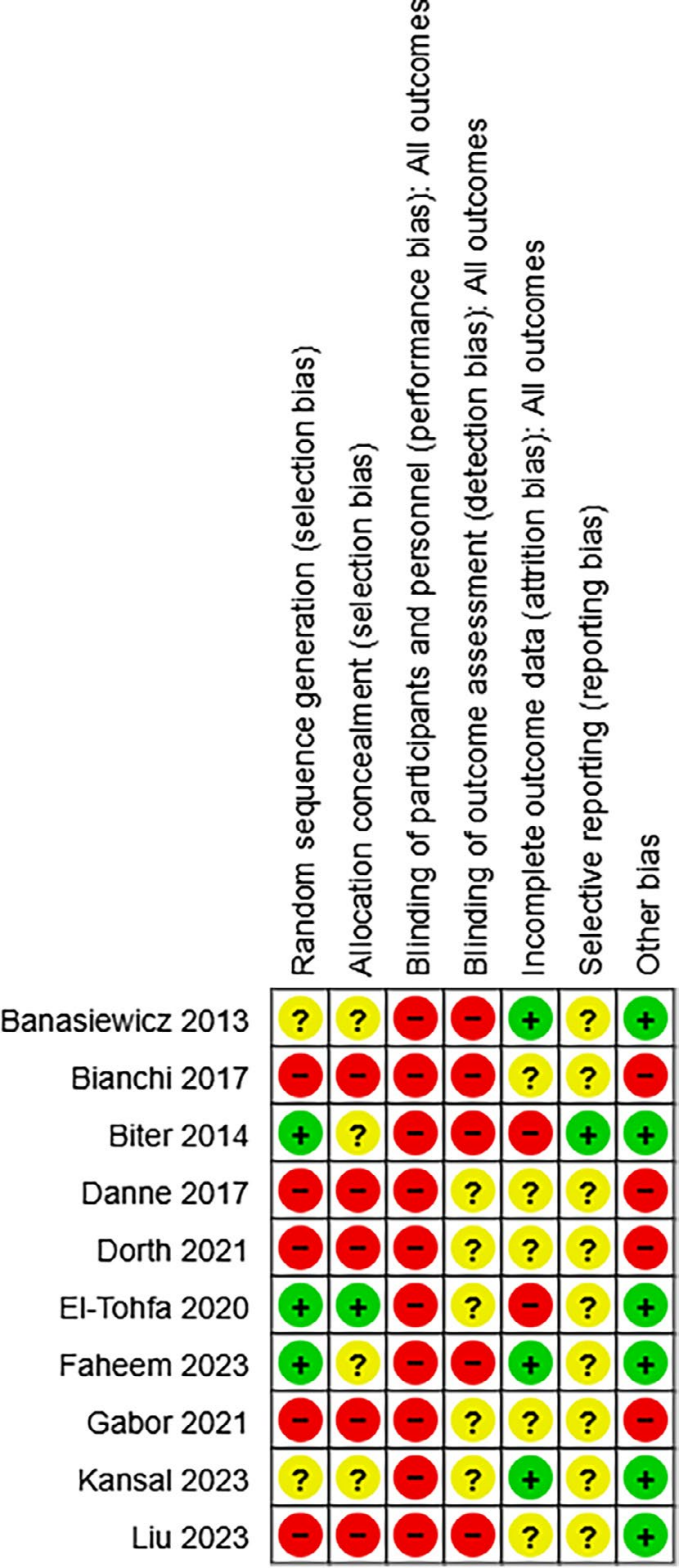
Review authors judgements about each risk of bias item for each included study.

The quality of studies was assessed independently by two authors, using the Cochrane Collaboration tool for assessing risk of bias [11]. This tool addresses six specific domains, namely sequence generation, allocation concealment, blinding, incomplete outcome data, selective outcome reporting and other bias (see Figure 1). We completed a ‘Risk of bias’ table for each eligible study. This was incorporated into an overall grading of the evidence related to each of the main outcomes using GRADE (Grades of Recommendation, Assessment, Development and Evaluation) [15]. The certainty of the body of evidence was assessed against five principle domains: 1. Limitations in design and implementation; 2. Indirectness of evidence or generalisability of findings; 3. Inconsistency of results, for example unexplained heterogeneity and inconsistent findings; 4. Imprecision of results where confidence intervals are wide; and 5. Publication bias [11].

## 3 Results

### 3.1 Results of the Search

**FIGURE 2:**
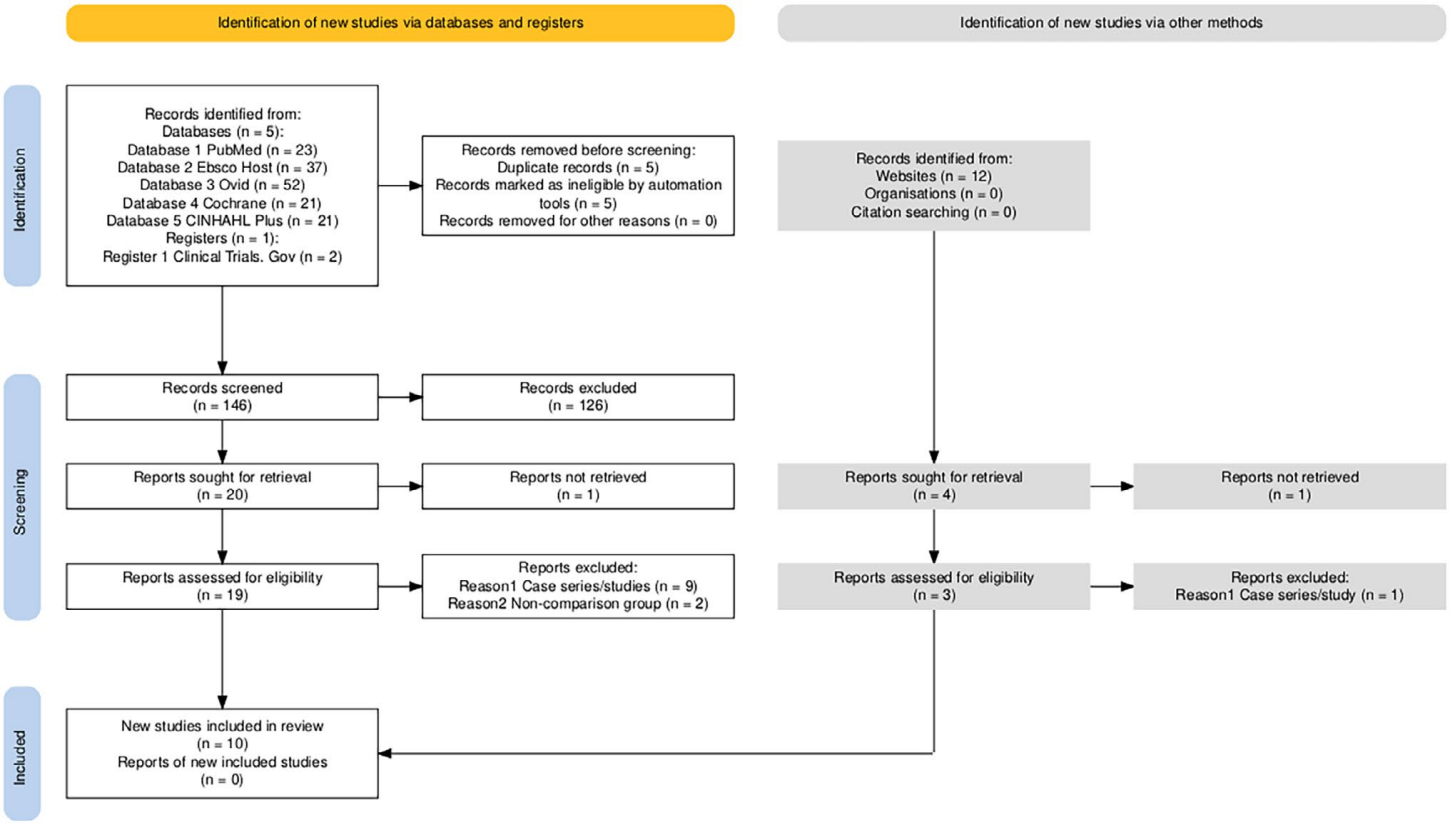
PRISMA 2020 flow diagram of the included studies.

As shown in Figure 2, following reviews of titles & abstracts from a total of 168 citations, 10 were found to be duplicates and were excluded and further 134 citations were excluded as they were not related to the systematic review question. A further 2 studies were requested but not retrieved. Subsequently, following a review of the full texts of the remaining 22 published papers, a further 12 studies were excluded (see Table 1). This left 10 studies [1, 9, 20, 21, 22, 23, 24, 25, 26, 27] which met the inclusion criteria.

**TABLE 1:** Excluded studies and reason.

Author	Reason for exclusion
Vaughn, Lalikos [16]	Case series
Lynch [17]	Case series
Irkoren [18]	Case study
Wang [19]	Case study
De Azevedo [37]	Case study
Bendewald [38]	Case series
Nakamichi [39]	Case series
Giordano [40]	No comparison group
Hannan [8]	No comparison group

### 3.2 Overview of the Included Studies

Table 2 provides an overview of the included studies.

**TABLE 2:** Characteristics of included studies.

Author	Country	Sample	Primary outcome	Secondary outcomes	Study type	Intervention	Comparison
Banasiewicz [20]	Poland	19	Time to healing	Pain	RCT	NPWT	Open wound with conventional dressing
Biter [21]	Netherlands	49	Time to healing	Recurrence rate Pain	RCT	NPWT	Open wound with silicone dressing and absorbing dressing
Bianchi [22]	USA	130	Not stated	Recurrence rate	Pre‐post study	NPWT	Closure incision; Treatment not stated
Danne [23]	Australia	62	Time to healing	Recurrence rate	Retrospective study	NPWT	Open wound with conventional dressing
El‐Tohfa [24]	Egypt	50	Time to healing	Recurrence rate	Prospective cohort study	NPWT	Open wound treated with Iruxol ointment and silver nitrate ointment
Dorth [26]	Germany	85	Not stated	Recurrence rate	Retrospective study	NPWT	Slide‐swing plasty; Open excision; Treatment not stated
Gabor [1]	Brazil	21	Time to healing	Pain	Retrospective study	NPWT	Closure incision with conventional dressing
Faheem [25]	Pakistan	50	Wound Size	Recurrence rate Pain	RCT	NPWT	Open wound with silicone dressing and absorbing dressing
Kansal [9]	India	81	Time to healing	Pain	Prospective cohort Study	NPWT	Open wound with conventional dressing
Liu [27]	China	62	Number wounds healed	Recurrence rate Pain Patients' experience	Retrospective Study	NPWT	Closure incision with conventional dressing

### 3.3 Study Designs

The included studies varied in design and were published between 2013 and 2023. Three studies [20, 21, 25] were RCT's and four studies [1, 23, 26, 27] were retrospective in nature. Two studies [9, 24] carried out prospective cohort studies and one study [22] undertook a pre‐post study.

### 3.4 Geographical Location

The selected studies were from all over the world, including Poland [20], Netherlands [21], India [9], Pakistan [25], Brazil [1], Australia [23], China [27], Egypt [24], Germany [26] and United States of America (USA) [22].

### 3.5 Study Settings

Apart from one study [23] in which the population was selected from a private surgical practice, all other 9 studies were based in a hospital setting [1, 9, 20, 21, 22, 24, 25, 26, 27]. One study [20] was based on outpatient's department of proctology and surgery, department of general surgery, surgery gastrointestinal oncology and plastic surgery. One study [26] selected their population from 2 departments of paediatric surgery.

### 3.6 Sample Size and Demographics

The overall sample sizes of the studies were relatively small ranging from 19 participants [20] to 130 participants [22], with an average size of 61 (SD: 33) participants, from a total of 609 participants. Within the 10 studies, 8 studies [1, 9, 21, 22, 23, 24, 26, 27] included male and female gender and two studies [20, 25] only included male. Overall, with a predominance of male gender of 76.6% (*n* = 467) and female 23.3% (*n* = 142). It was not possible to calculate the mean age of participants across all studies as some authors did not provide enough information. However the population in the studies varied, including children [26] with a mean range of 14.72, to older adults of 69 years old [23].

### 3.7 Intervention and Comparison

Two different wound closure techniques were observed within the 10 included studies. Table 2 outlines the intervention and comparison for wounds that were closed using sutures and for wounds that were left to heal by secondary intention. A total of 4 studies [1, 22, 26, 27] compared treatment of PS following primary wound closure and NPWT, whereas, 6 studies [9, 20, 21, 23, 24, 25] compared treatment with NPWT in open wounds. Two studies [1, 27] compared NPWT to a conventional dressing, one study [26] compared NPWT to slide‐swing plasty or open wound healing with no treatment stated. One study [22] compared NPWT versus closed incision with no treatment stated. Table 2 also shows one study [24] compared NPWT versus open wound treated with Iruxol ointment and silver nitrate ointment, two studies [21, 25] compared NPWT versus open wound treated with silicone dressing and an absorbent dressing, and three studies [9, 20, 23] compared NPWT and conventional dressing.

### 3.8 Follow‐Up

The follow‐up time for the primary outcome was defined as the duration of wound healing after excision of the PS.

### 3.9 Summary of Findings of Included Studies

As shown in Table 3, eight studies [1, 9, 20, 21, 23, 24, 25, 27] measured wound healing, seven studies [9, 21, 22, 23, 24, 26, 27] measured recurrence rates, six studies [1, 9, 20, 21, 25, 27] measured pain and one study [27] measured patients' experience. Table 3 also includes an overall grading of the evidence related to the outcomes using the GRADE approach [11, 15]. This approach defines the certainty of a body of evidence regarding the extent to which, one can be confident that an estimate of effect or association is close to the quantity of specific interest [15].

**TABLE 3:** Summary of findings of included studies.

Author	Results primary outcome	Results secondary outcomes			Certainty of evidence (Grade)
	Time to healing	Recurrence	Pain	Patient's experience	
Banasiewicz et al. [20]	Mean and SD (days): Intervention: 11.8 ± 4.7; Comparison: 30.3 ± 8.3	N/A	Mean and SD day 7: Intervention: 0.9 ± 0.7; Comparison: 2 ± 0.7	N/A	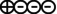 Very low^a^, ^c^
Biter et al. [21]	Median and range (days): Intervention: 84 (34–349); Comparison: 93 (43–264)	N (%): Intervention: 3 (13%); Comparison: 1 (1%)	Mean day 14: Intervention: 2.2; Comparison: 2.5	N/A	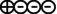 Very low^b^
Bianchi et al. [22]	N/A	N (%): Intervention: 2/65 (3%); Comparison: 8/65 (12%)	N/A	N/A	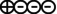 Very low^b^
Danne et al. [23]	Mean and SD (weeks): Intervention: 9.8 ± 6.3; Comparison: 15.0 ± 18.1	N (%) Intervention: 1 (3.1%); Comparison: 4 (12.5%)	N/A	N/A	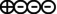 Very low^a^, ^b^
El‐Tohfa et al. [24]	Mean and SD (weeks): Intervention: 5.28 ± 0.92; Comparison: 8.55 ± 1.02	N (%): Intervention: 1 (4%); Comparison: 3 (15%)	N/A	N/A	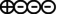 Very low^a^, ^b^
Dorth et al. [26]	N/A	N (%): Intervention: (9%) Comparison: (23%)	N/A	N/A	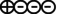 Very low^b^
Gabor et al. [1]	Median and Range (days): Intervention: 23.8 (14–28); Comparison: 57.9 (35–112)	N/A	Mean: day 7: Intervention: 0.90; Comparison: 2.63	N/A	N/A
Faheem et al. [25]	Wound size (mean and SD) at day 14: Intervention: 14.93 ± 9.14; comparison: 24.42 ± 9.85	N/A	Mean and SD day 14: Intervention: 1.24 ± 0.68; Comparison: 1.52 ± 1.56	N/A	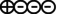 Very low^c^
Kansal et al. [9]	Mean and SD (days): Intervention: 59.24 ± 10.21; Comparison: 75.31 ± 14.68	N (%): Intervention *n* = 1 (0.02); comparison *n* = 1 (0.03)	Mean and SD day 14: Intervention: 2.29 ± 1.27; Comparison: 3.82 ± 1.55	N/A	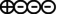 Very low^a^, ^b^, ^c^
Liu et al. [27]	Wounds healed (*n*; %): Intervention: 30 (93.75%); Comparison: 23 (76.67%)	N (%); Intervention: 0 (%); Comparison: 2 (6.67%)	Mean and SD 24 h after surgery: Intervention: 3.65 ± 0.27; Comparison: 4.29 ± 0.36	Mean and SD; Intervention: 6.94 ± 0.63; Comparison: 5.27 ± 0.84	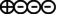 Very low^a^, ^b^, ^c^

*Note:* GRADE Working Group grades of evidence. High certainty: we are very confident that the true effect lies close to that of the estimate of the effect. Moderate certainty: we are moderately confident in the effect estimate: the true effect is likely to be close to the estimate of the effect, but there is a possibility that it is substantially different. Low certainty: our confidence in the effect estimate is limited: the true effect may be substantially different from the estimate of the effect. Very low certainty: we have very little confidence in the effect estimate: the true effect is likely to be substantially different from the estimate of effect.

^a^ Very low: Downgraded by study design, risk of bias and imprecision.

^b^ Very low: Downgraded by study design and risk of bias.

^c^ Very low: Downgraded by study design, risk of bias and heterogeneity.

### 3.10 Risk of Bias of the Included Studies

The risk of bias in the included studies is summarised in Figure 1. Two review authors independently assessed the risk of bias for each study and resolved any disagreements through consensus. For all included studies the Cochrane tool for assessing risk of bias was used [11].

### 3.11 Selection Bias

#### 3.11.1 Generation of the Randomisation Sequence

Two studies [21, 25] were judged to be at low risk of selection bias, because they described the use of robust randomisation methods for random sequence generation. Three studies [9, 20, 24] did not provide information about generation of randomisation sequence and therefore we judged these to be unclear risk of bias. The remaining five studies [1, 22, 23, 26, 27] did not provide information about generation of randomisation sequence as the methodology of studies did not include this domain, and we judged to be at high risk of bias.

#### 3.11.2 Allocation Concealment

Five studies [1, 22, 23, 26, 27] did not describe appropriate methods for generating the allocation sequence, and we judged these to be at high risk of bias in this domain. The remaining five [9, 20, 21, 24, 25] presented unclear methods for concealing group allocation thus we judged these to be at unclear risk of bias.

### 3.12 Performance Bias

#### 3.12.1 Blinding Participants and Personnel

All studies [1, 9, 20, 21, 22, 23, 24, 25, 26, 27] were judged at high risk of bias in this domain, as participants and personnel were not blinded due to the nature of the intervention.

### 3.13 Detection Bias

#### 3.13.1 Blinding of Outcome Assessment

Five studies [20, 21, 22, 25, 27] were judged to be at high risk of bias in this domain, as outcomes assessors were not blinded. Five of the studies [1, 9, 23, 24, 26] did not provide information on the blinding of the outcome assessors, and we judged these to be at unclear risk of bias.

### 3.14 Attrition Bias

#### 3.14.1 Incomplete Outcome Data

Three studies [9, 20, 25] were judges to be at low risk of bias in this domain, as they conducted intention to treat analysis. Five studies [1, 22, 23, 26, 27] did not provide information about generation of randomisation sequence as the methodology of studies did not include this domain, therefore, were judged to be at high risk of bias. The remaining two studies [21, 24] had drop‐outs which were not comprehensibly described with regard to the numbers of participants in the intervention group and the control group and therefore, we judged these to be at high risk of bias.

### 3.15 Reporting Bias

#### 3.15.1 Selective Reporting

Only one study [21] was judged to be at low risk of bias in this domain, as it reported planned outcomes alluded to in the paper. Remaining nine studies [1, 9, 20, 22, 23, 24, 25, 26, 27] were judged to be at unclear risk of reporting bias, as it was unclear whether all planned outcomes were reported.

### 3.16 Other Bias

Six studies [9, 20, 21, 24, 25, 27] were judged to be at low risk of bias in this domain, as other potential sources of bias were not identified. The remaining four studies [1, 22, 23, 26] were judged as high risk of bias due to the nature of the methodology of studies.

### 3.17 Results for the Primary Outcome: Wound Healing

Table 3 provides an overview of the results for the primary outcome, wound healing (time to healing).

#### 3.17.1 Wound Healing in Days

**FIGURE 3:**
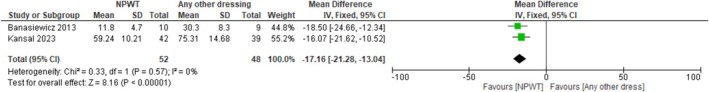
Forest plot of wound healing in days comparing NPWT versus any other dressing.

Figure 3 outlines the results for the meta‐analysis of 2 studies [9, 20], measuring wound healing in days (mean; SD). NPWT may reduce the mean time to wound healing in days compared to any other dressing, but the evidence is very uncertain, [MD: −17.16 days (95% CI −21.28 to −13.04; *p* < 0.00001), very low certainty evidence: downgraded for study design, risk of bias and imprecision].

#### 3.17.2 Wound Healing in Weeks

**FIGURE 4:**
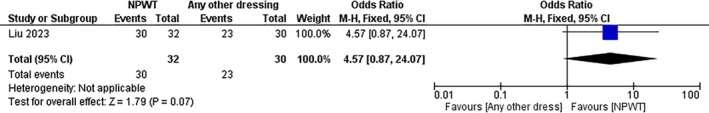
Forest plot of wound healing in weeks comparing NPWT versus any other dressing.

Figure 4 outlines the results for the meta‐analysis of 2 studies [23, 24] measuring wound healing in weeks (mean; SD). NPWT may reduce the mean time to wound healing in weeks compared to any other dressing, but the evidence is very uncertain [MD: −3.28 weeks (95% CI −3.82 to −2.75; *p* < 0.00001), very low certainty evidence: downgraded for study design, risk of bias and imprecision].

#### 3.17.3 Wound Healing‐Median and Range

One study [21] reported healing time was on average of 84 days for the NPWT group and 93 days for the conventional dressing group. Indicating a median reduction in the healing time of 9 days in the NPWT group compared to silicone and absorbing dressings. Another study, Median healing time in the NPWT group was 23.8 days (14–28) while this was 57.9 days (35–112) for the comparison group [1]. This indicates a median reduction in the healing time, of 34.1 days in the NPWT group compared with the conventional dressings group.

#### 3.17.4 Wound Size at the End of the Study

In one study [25] the wound size was explored and compared for both groups at baseline and on day 14. At baseline, the mean wound size for the intervention group was 30.97 [2] and 36.33 cm [2] for the comparison group, and on day 14 the mean wound size for the intervention group was 14.93 cm [2] and 24.42cm [2] for the comparison group [25]. A reduction in wound size by 16.04 cm [2] was reported when using NPWT and a reduction in wound size by 11.91 cm [2], was reported for the conventional dressing group [25].

#### 3.17.5 Number of Wounds Healed

**FIGURE 5:**
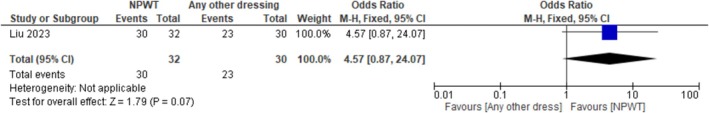
Forest plot of wounds healed comparing NPWT versus any other dressing.

Figure 5 outlines the results of one study [27] on the number of wounds healed at the end of the study. NPWT may have little to no effect on the number of wounds healed compared to any other dressings, but the evidence is very uncertain, [30 patients (93.75%) healed in the NPWT group while 23 patients (76.67%) healed in the comparison group, OR: 4.57, 95% CI: 0.87 to 24.07; *p* = 0.07, very low certainty of evidence downgraded for study design, risk of bias and imprecision].

### 3.18 Results for the Secondary Outcomes

Table 3 provides an overview of the results for the secondary outcomes: recurrence, pain and patient's experience.

#### 3.18.1 Recurrence

**FIGURE 6:**
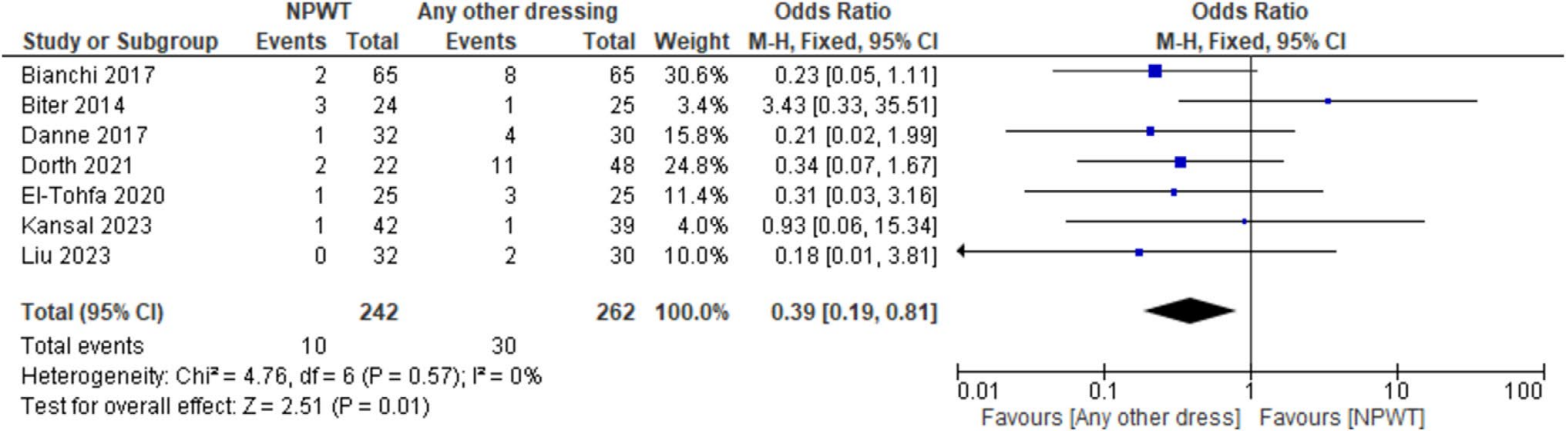
Forest plot of recurrence rate comparing NPWT versus any other dressing.

Figure 6 outlines the results for the meta‐analysis of seven studies [9, 21, 22, 23, 24, 26, 27], measuring the recurrence (OR). In the NPWT group 4% (10/242) developed recurrence, whereas in the any other dressing group 11% (30/242) developed recurrence. NPWT may reduce the odds of recurrence by 61% when compared to any other dressings, but the evidence is very uncertain, [OR: 0.39 (95% CI 0.19 to 0.81; *p* = 0.01), very low certainty of evidence downgraded for study design and risk of bias].

#### 3.18.2 Pain

**FIGURE 7:**
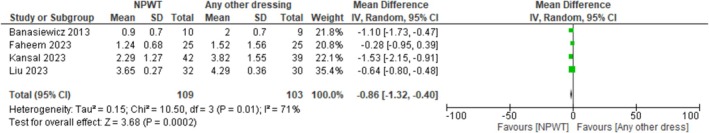
Forest plot of pain comparing NPWT versus any other dressing.

Six studies [1, 9, 20, 21, 25, 27] reported postoperative pain. The visual analogue scale (VAS) was used to report pain in all the six studies, which documents the severity of pain on a scale of 0 to 10. Data were entered into RevMan for four studies [9, 20, 25, 27] and results (mean; SD) are shown in Figure 7. Due to heterogeneity (*I* ^2^ = 71%), a random effects model was employed. Patients using NPWT may experience less severe pain compared to any other dressing, but the evidence is very uncertain, [MD: −0.86 (95% CI −1.32 to −0.40, *p* < 0.00002), very low certainty evidence: downgraded for study design, risk of bias and heterogeneity].

One study [21] reported median pain at day 14 of 2.2 for the NPWT group and 2.5 for the dressing group. One study [1] reported median pain at day 7 of 0.9 for the NPWT group and 2.63 for the dressing group. Both studies indicating that patients experienced less severe pain when using NPWT.

#### 3.18.3 Patients' Experience

**FIGURE 8:**
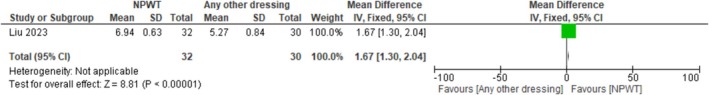
Forest plot of patients' experience comparing NPWT versus any other dressing.

Figure 8 outlines the results for one study [27] which reported patients' experience (mean; SD) using a self‐made satisfaction scale, ranging from 0 to 10 points, whereby higher scores indicate greater levels of patients' satisfaction. The mean score for the NPWT group was 6.94 ± 0.63 compared to 5.27 ± 0.84 for the conventional therapy group [27]. NPWT may provide greater levels of patient's satisfaction compared to any other dressing, but the evidence is very uncertain, [MD: 1.67 (95% CI: 1.30 to 2.04), *p* = 0.0001, with very low certainty evidence: downgraded for study design and risk of bias].

## 4 Discussion

The main goal of this systematic review was to explore the impact of NPWT on healing in patients post excision of PS, within which the primary outcome was wound healing rate. Ten articles were included, with a total of 609 participants and mean sample size of 61 (SD: 33). Two of the included studies [22, 26] did not analyse the primary outcome and one study [25] proposed to study time of healing but failed to do so and reported wound size instead.

PS is a very common disease with an incidence rate of 26 per 100 000 patients per year, predominantly in young males with a ratio of male to female of 3–4:1 [28]. The findings within this systematic review are in line with this epidemiology with an incidence predominantly in males, however it was not possible to ascertain the age mean range across all studies. The treatment approach for symptomatic PS typically involves surgical intervention, with complete excision of the abscess, however it is still controversial whether to close the wound or leave it open for secondary healing [29]. The ideal approach for PS is one that is easily reproducible while is tolerated by the patient and successfully addresses the underlying disease, promoting wound healing with a low recurrence rate [30].

This systematic review explored healing by both, primary and secondary intention using NPWT versus any other dressings. Findings from 6 studies with NPWT (secondary intention) [9, 20, 21, 23, 24, 25] suggest quicker healing time when compared to any other dressings. One study with closure and NPWT [1] showed reduction in wound size when using NPWT in comparison with any other treatment. However, the body of evidence was assessed as being very low certainty; thus, it is very uncertain whether NPWT makes any difference in wound healing time.

One study [27] did not find statistically significant difference in the number of wounds healed, thus NPWT may have little to no effect on the number of wounds healed compared to any other dressings. Time of healing was not reported which could have been an indicator of treatment efficiency. Nonetheless, the body of evidence was assessed as being very low certainty, thus, it is very uncertain whether NPWT makes any difference in the number of wounds healed.

Evidence shows that PS wounds heal faster after primary closure than after open healing, yet at the expense of increased risk of recurrence [31]. However, the use of NPWT for complex wounds has been shown to be effective in reducing recurrence rates [5, 32]. Seven studies analysed recurrence rate [9, 21, 22, 23, 24, 26, 27] and apart from one study [21] all others demonstrated a possible reduction in the odds of recurrence in favour of NPWT, regardless of whether healing by primary or secondary intention. However, the body of evidence was assessed as being very low certainty, thus, it is very uncertain whether NPWT makes any difference in reducing the odds of wound recurrence.

Literature suggests that one of the most common complications associated with NPWT is pain [33]. In this SR, six studies [1, 9, 20, 21, 25, 27] reported postoperative pain and findings show that NPWT may reduce the severity of pain compared to any other dressing, in line with the findings of a systematic review on the use of NPWT in comparison to conventional dressings [34]. However, the body of evidence was assessed as being very low certainty; thus, it is very uncertain whether patients using NPWT experience less pain compared to any other dressings.

Another outcome of this systematic review was to explore the patients experience with the use of NPWT in comparison with any other dressing. Patients' experience is essential when considering the appropriate wound dressing, as the chosen intervention may impact on a patient's quality of life [35]. Though, the evidence on the patients' experience in using NPWT for wound healing is spare [34]. This is also in line with the findings in this systematic review, where only one study [27] intentionally explored patient's satisfaction. Findings suggest that NPWT may provide greater levels of patient's satisfaction compared to any other dressing. However, the body of evidence was assessed as being very low certainty; thus, it is very uncertain whether NPWT provides greater levels of patient satisfaction.

In clinical practice, optimal healing treatment remains controversial and NPWT is not the panacea, with some patients reporting bad odour, noise, leakage and skin irritation [21]. Furthermore, for NPWT to be effective it must be applied by trained staff [24] and its application to the natal cleft of the buttocks can be challenging [20]. Additionally, the staff can encounter a shortage of NPWT supplies and tools [9] and the costs associated with its application are higher than conventional dressing [26]. Nevertheless, in the long‐term the effectiveness of NPWT reduces hospital stay, treatment costs and resources [36].

The cumulative evidence from the included studies supports the possible efficacy of NPWT in promoting faster wound healing, reducing the risk of recurrence, alleviating pain and enhancing patient satisfaction compared to other dressings. Though, the body of evidence was assessed and judged as being very low certainty, therefore, it is very uncertain whether NPWT makes any difference to any of the outcomes reported.

## 5 Limitations

Clinical research on interventions for wound healing is fraught with challenges. Evidence shows that internal validity is weak in wound RCTs, due to substantial heterogeneity in a multiplicity of confounding factors [16]. This was also observed in this systematic review. Moreover, the authors acknowledge that the variations in study design may affect the reliability of results and contribute to lower certainty, thus the findings should be interpreted with caution [17]. Critically, the selected studies were limited by a relatively small sample size which means that findings cannot be generalised [18]. The authors recommend larger scale studies (sample sizes), with accurate sample size calculation for the certain target population, to determine whether these outcomes can be applied to the general population.

### 5.1 Potential Biases in the Review Process

All efforts were made to limit potential biases in the review process, by implementing a systematic and rigorous, transparent and reproducible methodology [19]. As this systematic review was restricted to the English language, we cannot rule out that additional relevant studies may have been excluded. We recognise that this limitation may have excluded relevant research conducted in other languages, however, this was due to limited resources and language proficiency. All the included studies were judged as having an unclear or high risk of bias due to a lack of detail regarding sequence generation and for allocation concealment. Due to the nature of the intervention, dressing trials were not able to blind participants or personnel, thus, all the included studies were judged as having a high risk of bias (a somewhat expected result). All the included studies were judged as having an unclear or high risk of bias due to a lack of detail regarding outcome blinding outcome assessment. Two of the included studies did not analyse participants randomised to the intervention, and we therefore judged these to be at high risk of attrition bias and five as unclear due to lack of information. Nine out of the 10 studies were judged as high risk for selective reporting. Four of the selected studies were judged as high risk of other bias. Additionally, data surrounding benefits shown very low certainty of evidence, downgraded by study design, risk of bias, imprecision and heterogeneity, requiring cautious interpretation and demanding for more rigorous research.

## 6 Conclusion

This systematic review aimed to assess the impact of NPWT on healing in patients following excision of PS. Overall, the certainty of the evidence for all outcomes was very low, therefore it is unclear whether NPWT makes any difference to time to healing, pain, recurrence and patient's experience. The included studies were downgraded twice due to high risk of bias in numerous domains. Therefore, findings must be interpreted with caution, highlighting the need for more rigorous research. Further high‐quality research with larger sample sizes is recommended to clearly explore the impact of NPWT on PS healing, recurrence, pain and patient's satisfaction.

## Disclosure

This systematic review was conducted as part of the requirements for a Master's Degree and, therefore, was not registered.

## Conflicts of Interest

The authors declare no conflicts of interest.

## Data Availability

Given that this is a systematic review, there is no requirement for a data availability statement to be provided.
