# Correction to “What Is the Impact of Negative Pressure Wound Therapy on Healing in Patients Post Excision of Pilonidal Sinus? A Systematic Review and Meta‐Analysis”

**DOI:** 10.1111/iwj.70696

**Published:** 2025-05-26

L. P. F. Morais, Z. E. H. Moore, D. Patton, T. O. Connor, and H. J. E. Wilson, “What Is the Impact of Negative Pressure Wound Therapy on Healing in Patients Post Excision of Pilonidal Sinus? A Systematic Review and Meta‐Analysis,” *International Wound Journal* 22 (2025): e70194, https://doi.org/10.1111/iwj.70194.

Pinar Avsar has been added to the author list and author affiliations have been corrected.

Liliana Patricia Ferreira Morais^1,2^ | Zena Elizabeth Helen Moore^1,2,3,4,5,6,7,8^ | Declan Patton^1,2,3,4,5,9^ | Tom O' Connor^1,2,3,4,5,10^ | Pinar Avsar^1,2,3^ | Hannah Jane Elizabeth Wilson^1,2,3^

1. Royal College of Surgeons in Ireland (RCSI), University of Medicine and Health Sciences, Dublin, Ireland

2. School of Nursing and Midwifery, RCSI University of Medicine and Health Sciences, Dublin, Ireland

3. Skin Wounds and Trauma Research Centre, RCSI University of Medicine and Health Sciences, Dublin, Ireland

4. Fakeeh College of Health Sciences, Jeddah, Saudi Arabia

5. School of Nursing and Midwifery, Griffith University, Brisbane, Queensland, Australia

6. Department of Public Health, Faculty of Medicine and Health Science, Ghent University, Shanghai, China

7. National Health and Medical Research Council Center of Research Excellence in Wiser Wound Care, Menzies Health Institute Queensland, Brisbane, Queensland, Australia

8. Honorary Visiting Professor, Cardiff University, Cardiff, Wales, UK

9. Honorary Senior Fellow, Faculty of Science, Medicine and Health, University of Wollongong, Wollongong, New South Wales, Australia

10. Lida Institute, Shanghai, China

We apologise for this error.

